# Dining dichotomy: aquatic and terrestrial prey capture behavior in the Himalayan newt *Tylototriton verrucosus*

**DOI:** 10.1242/bio.020925

**Published:** 2016-09-09

**Authors:** Egon Heiss, Marie De Vylder

**Affiliations:** 1 Istitute of Systematic Zoology and Evolutionary Biology, Friedrich-Schiller-University of Jena, Jena, Germany. Erbertstr, Jena 1 07743, Germany; 2 Department of Biology, University of Antwerp, Antwerp, Belgium. Universiteitplein 1, Antwerp B-2610, Belgium

**Keywords:** Amphibians, Feeding, Kinematics, Behavioral flexibility

## Abstract

Transitions between aquatic and terrestrial prey capture are challenging. Trophic shifts demand a high degree of behavioral flexibility to account for different physical circumstances between water and air to keep performance in both environments. The Himalayan newt, *Tylototriton verrucosus*, is mostly terrestrial but becomes aquatic during its short breeding period. Nonetheless, it was assumed that it lacks the capability of trophic behavioral flexibility, only captures prey on land by its tongue (lingual prehension) and does not feed in water. This theory was challenged from stomach content analyses in wild populations that found a variety of aquatic invertebrates in the newts' stomachs during their breeding season. Accordingly, we hypothesized that *T. verrucosus* actively changes its terrestrial prey capture mechanism to hunt for aquatic prey at least during its aquatic stage. In fact, the kinematic analyses showed that *T. verrucosus* uses lingual prehension to capture prey on land but changes to suction feeding for aquatic strikes. The statistical analyses revealed that terrestrial and aquatic strikes differ significantly in most kinematic parameters while behavioral variability does not differ between both behaviors. In turn, the movement patterns in suction feeding showed a higher degree of coordination between jaw and hyoid movements compared to the putative primary feeding mode, namely lingual prehension. We conclude that *T. verrucosus*, though relatively slow compared to trophic specialists, benefits from a high degree of behavioral flexibility that allows exploiting food sources efficiently from two very different habitats.

## Abstract (precis)

**Summary:** *Tylototriton verrucosus* uses lingual prehension for terrestrial prey capture and switches to suction feeding for aquatic strikes. This flexible prey capture behavior enables efficient food acquisition in two very different habitats.

## INTRODUCTION

Salamanders can capture prey in aquatic and terrestrial habitats. Most salamanders are only specialized to one environment, but few species can exploit food sources both from aquatic and terrestrial domains (Deban and Wake, 2000; Miller and Larsen, 1990, 1989; Wake and Deban, 2000). The main challenge of trophic habitat switches are the different demands on the prey capture apparatus due to the physical differences between water and air, such as differences in density and viscosity (Bramble and Wake, 1985; Lauder, 1985). In fact, there are only a few vertebrates that can handle the challenges associated with a switch between media to capture prey in both environments (e.g. Deban and Marks, 2002; Heiss et al., 2013a, 2015; Lauder and Shaffer, 1988; Michel et al., 2015a,b; Natchev et al., 2010, 2015; Reilly, 1996; Stayton, 2011; Van Wassenbergh, 2013; Van Wassenbergh et al., 2006). When animals feed in both environments they can use the same set of movements, however they will perform suboptimally in at least one of the two environments; alternatively they can alter their feeding behavior to increase efficiency (Bramble, 1973; Stayton, 2011).

The main capture mode of salamanders in water is suction feeding, while on land a jaw- or tongue-based mechanism is used. In suction feeding, a fast oropharyngeal volume expansion draws prey and surrounding water to flow into the gaping mouth (Alexander, 1974; Lauder, 1985; Muller and Osse, 1984; van Leeuwen and Muller, 1984). For terrestrial capture events, salamanders have to account for the low viscosity and density of air and use their quickly protruded tongue to catch and bring prey into the mouth (lingual prehension), or grasp prey directly by their jaws (jaw prehension) (Bramble and Wake, 1985; Heiss et al., 2013a, 2015; Miller and Larsen, 1990; Wake and Deban, 2000). Suction feeding and lingual prehension are often regarded to represent the most effective capture modes in the respective medium, but suction feeding and lingual prehension rely on different sets of well-coordinated movements and require different – and often conflicting – morphological and functional adaptations of the hyobranchial apparatus (Deban, 2003). For example, while a robust hyobranchial system is advantageous to redirect muscular forces for the fast oropharyngeal volume expansion in suction feeding, a lightweight and flexible hyobranchial apparatus allows fast protrusion of the tongue for lingual prehension. As a consequence of this functional conflict, only few species have overcome the dichotomy and can employ both suction feeding and lingual prehension in an efficient way due to a morpho-functional bias towards one of the two capture modes.

In fact, some groups within extant salamanders are the only vertebrates known so far to switch between suction feeding and lingual prehension, but the efficiency of prey capture can vary substantially between those groups (Beneski et al., 1995; Deban and Marks, 2002; Heiss et al., 2013a, 2015; Lauder and Shaffer, 1988; Miller and Larsen, 1990, 1989; Reilly, 1995, 1996). Nevertheless, animals might greatly benefit from exploiting food sources from two very different environments where food abundance can differ substantially throughout the year. Some ambystomatid salamanders, for example, use suction in water but capture prey on short distances on land by their fleshy tongue (Beneski et al., 1995; Lauder and Shaffer, 1988). Other salamanders, such as some European newts, go a step farther and seasonally switch between an aquatic and a terrestrial habitat, undergoing seasonal morphological changes (Griffiths, 1997; Matthes, 1934; Thiesmeier and Schulte, 2010). These morphological changes result in distinct aquatic and terrestrial morphotypes (Heiss et al., 2016) that result in advanced aquatic and terrestrial prey capture performance, respectively (VanWassenbergh and Heiss, 2016). Seasonal habitat switches associated with morphological changes are certainly an aberrant feature within newts. Little is known regarding other newts that are merely terrestrial but invade water during the breeding season, without undergoing morphological changes. *Tylototriton verrucosus*, a representative of a basal clade within extant newts (Pyron and Wiens, 2011), is known to capture prey on land by lingual prehension but was hypothesized to lack the ability to feed in water (Miller and Larsen, 1989), though spending a reasonable time in aquatic realms during its breeding season (Dasgupta, 1996; Thorn, 1968). Interestingly, analyses of stomach contents in *T. verrucosus* revealed a considerable amount of consumed aquatic organisms (Dasgupta, 1996) and consequently it is likely that they capture prey in water at least during the breeding season. Accordingly, we hypothesize that *T. verrucosus* is capable of behavioral flexibility, actively adapting the primary lingual-based terrestrial prey-capture mode to aquatic demands and using a feeding mode based on jaw prehension or suction to catch prey in water. Here, we test this hypothesis by inducing *T. verrucosus* to feed in water, thus comparing the movement pattern used in terrestrial and aquatic prey capture events and testing for differences in behavioral variation and coordination of movements between feeding modes.

## RESULTS

All tested animals readily fed both in terrestrial and aquatic trials but the cranio-cervical movement patterns differed between terrestrial and aquatic capture events. Accordingly terrestrial and aquatic feeding are described separately.

### Terrestrial feeding

**Fig. 1.**
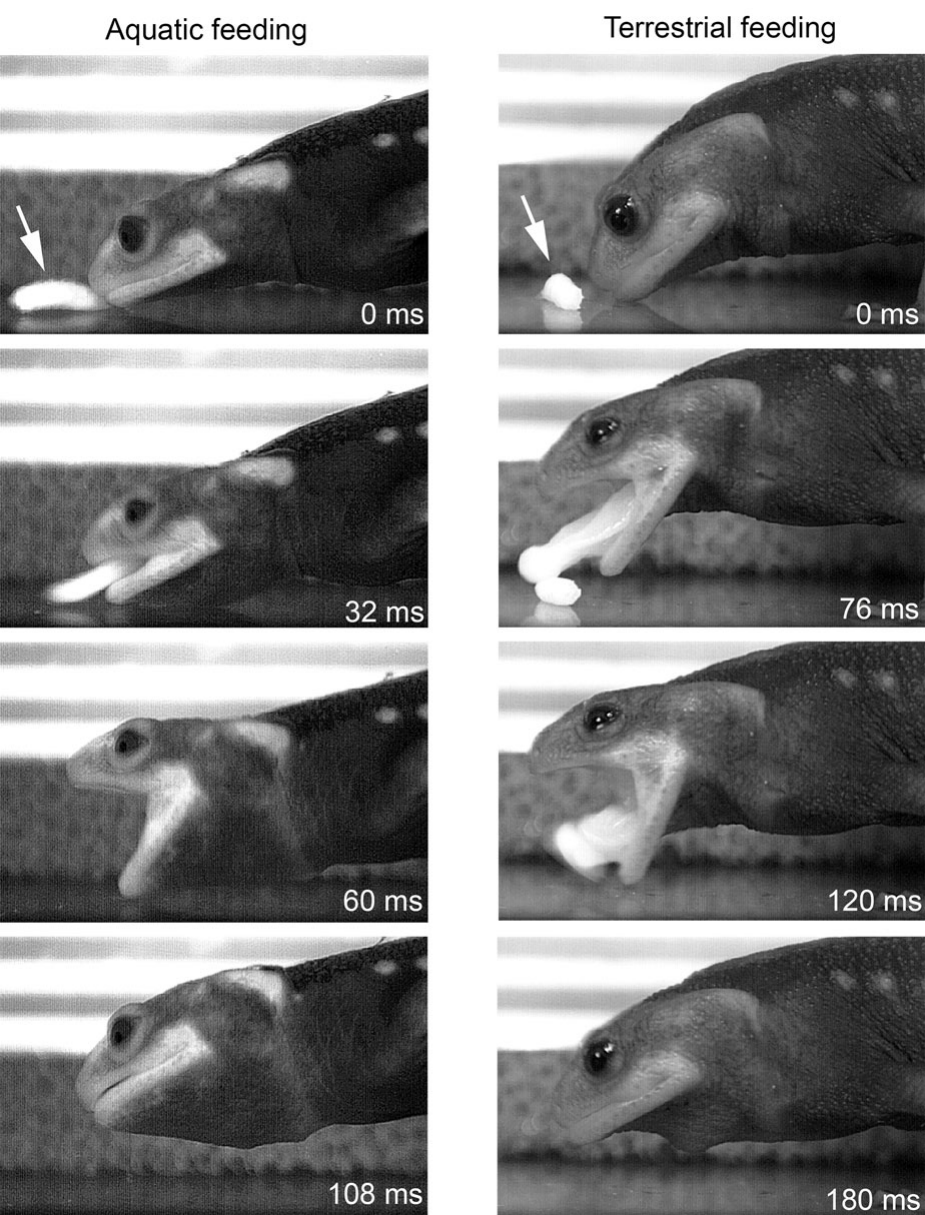
**Selected frame shots from high-speed recordings showing aquatic (left) and terrestrial (right) capture of prey in *T. verrucosus*.** Note that in water, *T. verrucosus* uses suction feeding while lingual prehension on land. The arrow indicates the prey (maggot).

The newts readily responded to the maggots offered with forceps ∼2 cm in front of the snout by directing their head towards the maggot and fixing it. The newts only considered wriggling maggots, while immobile maggots were ignored. The newts approached their (wriggling) prey up to a distance of ∼1 cm and then captured it by their quickly protruded adhesive tongue that was immediately retracted to bring the adhering maggot into the mouth (Fig. 1). The capture event was occasionally accompanied by a quick lunge of the whole body towards the prey with gaping mouth, but prey was always captured by the tongue.

**Fig. 2.**
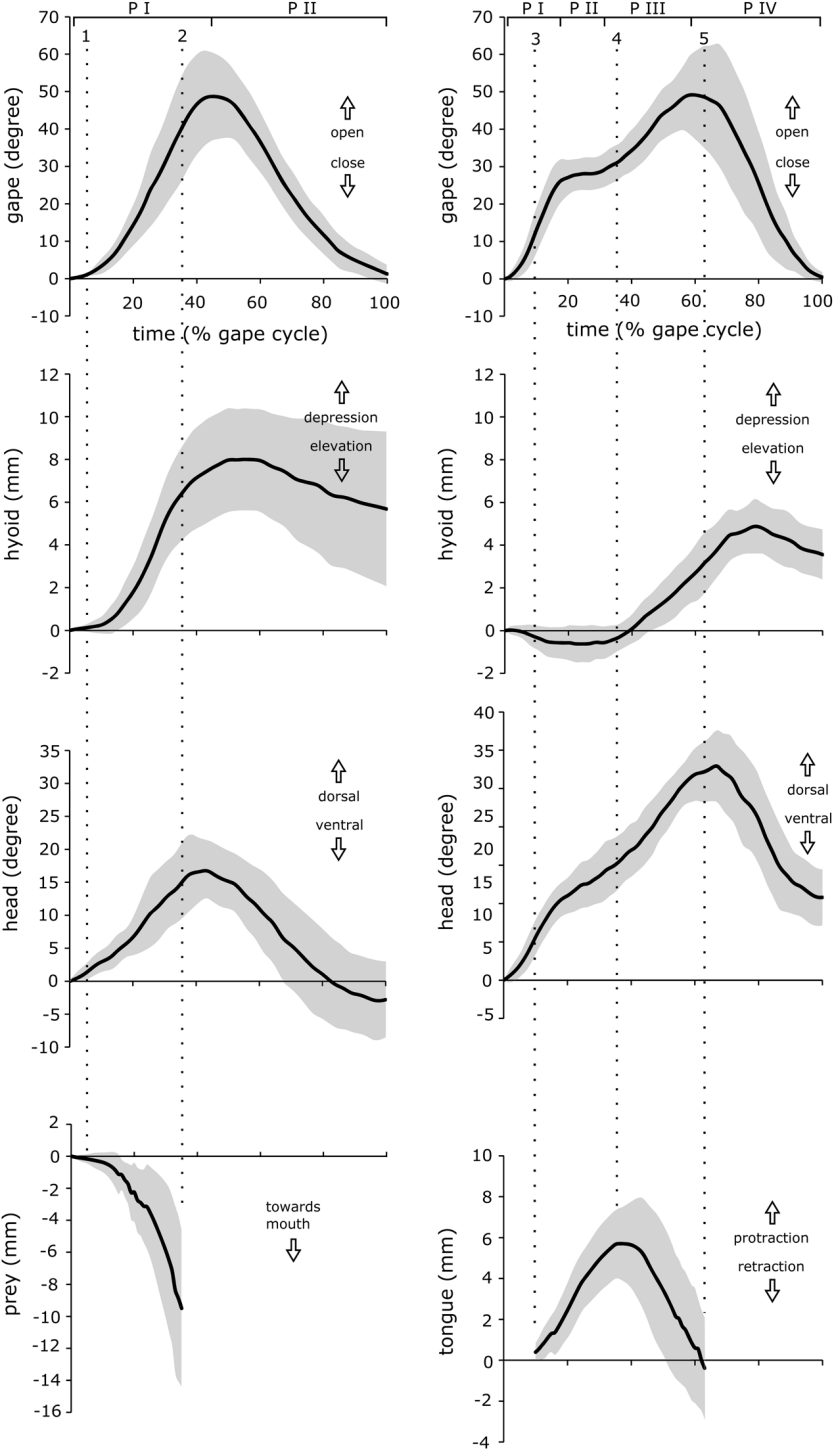
**Kinematic profiles showing gape, hyoid, head, prey and tongue movements in suction feeding (left) and lingual prehension (right).** Movements of prey are only shown in suction feeding while tongue movements only in lingual prehension. The gape cycle in suction feeding can be subdivided into two distinct phases (PI-PII), while the gape cycle in lingual prehension can be subdivided into four distinct phases (PI-PIV). The dashed lines indicate start (1) and end (2) of prey movement towards the salamander's mouth in suction feeding as well as start of tongue protraction (3), maximal tongue extension (4) and tongue retraction (5) in lingual prehension. Values: mean±s.d. (*n*=30).

The mean normalized kinematic profiles for jaw, hyoid, head and tongue movements are shown in Fig. 2. A whole gape cycle, defined as start of mouth opening until mouth closure, lasted 168±21 ms (mean±s.d.). The average kinematic profile of the gape described a cycle that, according to the course of its curve, could be subdivided into four phases. Start and end of each phase was defined as an abrupt change in the kinematic gape profile (see Fig. 2). Mouth opening comprised the first three phases and lasted 97±17 ms, and mouth closing the fourth phase which lasted 70±15 ms (Fig. 2). In the first phase, the mouth was quickly opened, while the hyoid was elevated and the tongue started protracting out of the mouth (Fig. 2). In the second phase, gape opening was rapidly decelerated, resulting in a short plateau-like profile. After the plateau-like second phase, the third phase started as mouth opening was accelerated again until gape reached its peak angle of 54±12 degrees. At the start of the third phase, hyoid depression started and with a short delay, the tongue protrusion reached its peak and contacted the prey. After prey contact, tongue with adhering prey started retracting. After peak gape (end of third phase), mouth closing defined the fourth gape-phase. In the fourth phase, hyoid depression reached its peak of 6.1±1.4 mm, tongue was retracted back into the mouth and prey was engulfed as mouth was closed.

### Aquatic feeding

In water, newts appeared more active and vigorously reacted to the offered maggots. Once detected, the prey was quickly approached and engulfed by a fast suction strike. The whole capture event lasted 113±19 ms and the gape profile described a bell-like shaped curve with two distinct phases: mouth opening (phase one) and mouth closure (phase two). Mouth opening was slightly faster than mouth closing (Table 1). At the onset of gape opening the hyoid started depressing, and with a short delay, prey started to move towards the newts' mouth. The gape reached its peak of 52.4±10.9 degrees after 51.7±7.6 ms, after which the second phase started and mouth was closed within 61.3±15.4 ms. Shortly after maximum gape, hyoid depression reached its ventral-most deflection and was slowly elevated again. Prey started to move towards the newts' mouth shortly after the onset of gape opening and hyoid depression. Prey was completely engulfed before both gape and hyoid reached their peaks (Fig. 2).

**Table 1.**
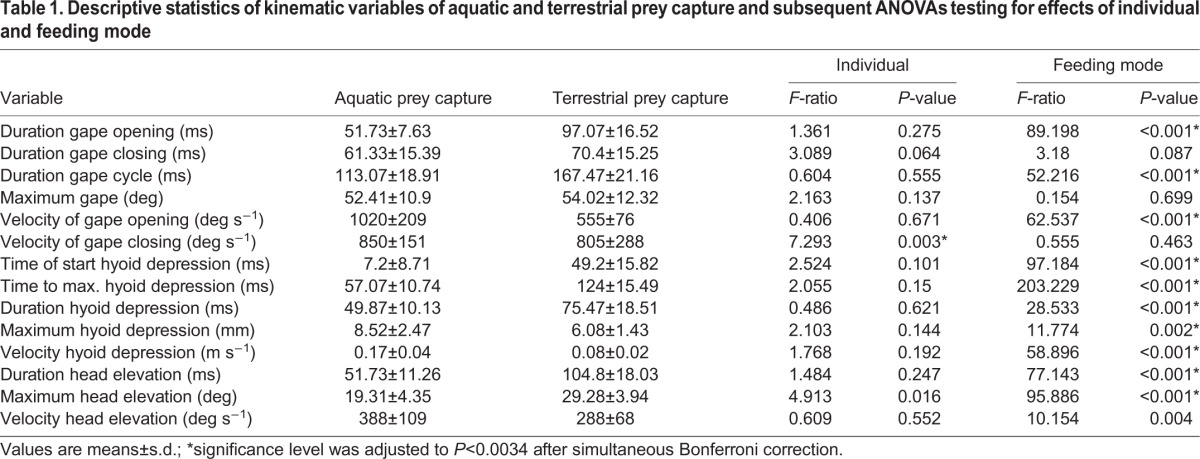
**Descriptive statistics of kinematic variables of aquatic and terrestrial prey capture and subsequent ANOVAs testing for effects of individual and feeding mode**

### Statistics

#### Differences between feeding modes (behavioral flexibility) and between individuals.

The MANOVA revealed significant differences between feeding modes (Wilks' lamda *F*_13, 12_=47.028, *P*<0.001) and between individuals (Wilks' lamda *F*_26, 24_=2.919, *P*=0.005). The subsequent series of ANOVAs revealed that the significant difference between feeding modes was based on significant differences in 10 out of the 14 variables tested. In contrast, the significant difference between individuals was based on significant differences of only one variable (Table 1).

Because of a significant interaction effect in the MANOVA between feeding mode and individual (Wilks' lamda *F*_26, 24_=2.712, *P*=0.008), subsequent *post hoc* tests were performed with Bonferroni correction. Pairwise comparison revealed individual differences when comparing aquatic and terrestrial strikes regarding the following variables: (i) velocity of gape closing differed significantly between aquatic and terrestrial strikes in individual 1 (*P*=0.036), individual 2 (*P*=0.024) but not in individual 3 (*P*=0.108); (ii) duration of hyoid depression differed significantly in individual 1 (*P*<0.001) and individual 3 (*P*=0.001) but not in individual 2 (*P*=0.634); (iii) maximum hyoid depression differed significantly in individual 2 (*P*=0.004) but not in the individuals 1 (*P*=0.176) and 3 (*P*=0.17); (iv) velocity of head elevation differed significantly in individual 3 (*P*=0.001) but not in individuals 1 (*P*=0.597) and 2 (*P*=0.269).

**Fig. 3.**
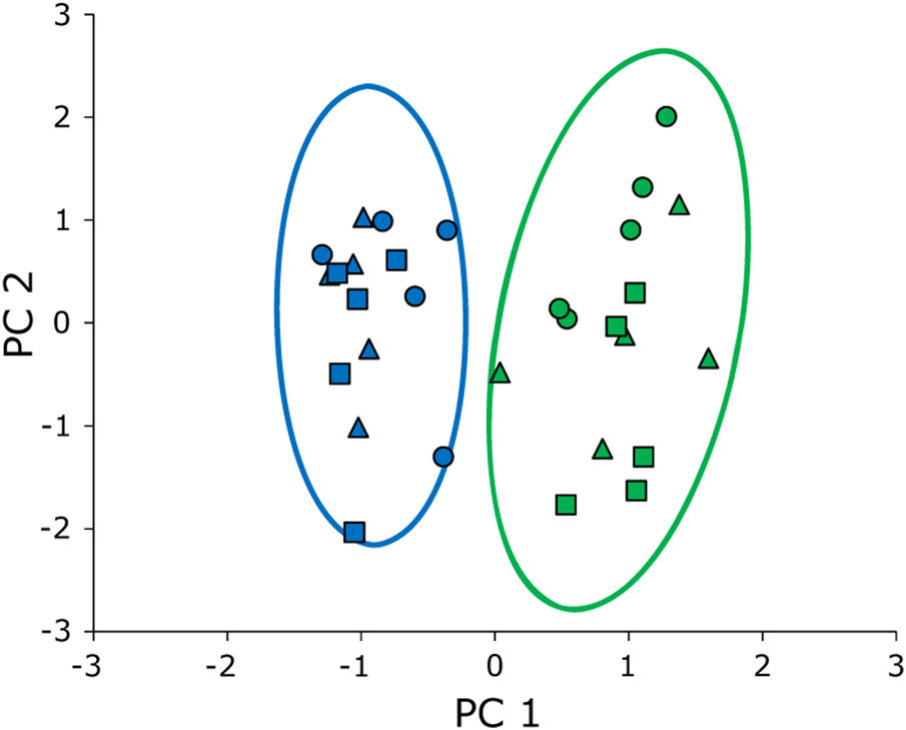
**Scatterplots of the first two principal components (PC1 and PC2) derived from the 14 kinematic variables to illustrate the multivariate relationship between the two feeding modes.** Blue, aquatic suction feeding; green, terrestrial lingual prehension; individuals indicated by different symbols. The ellipses display 95% confidence interval of the respective feeding mode. PC1 explains 54.9% and PC2 16.8% of the total variance.

Dispersion of kinematics among the two feeding modes and the three individuals on the first two principal component axes are shown in Fig. 3, and the loadings of the kinematic variables on the first three principal components are given in Table 2. While the two feeding modes (aquatic suction feeding and terrestrial lingual prehension) are well separated in kinematic space with no overlap, individuals show a similar distribution pattern (indicated by different symbols in Fig. 3).

**Table 2.**
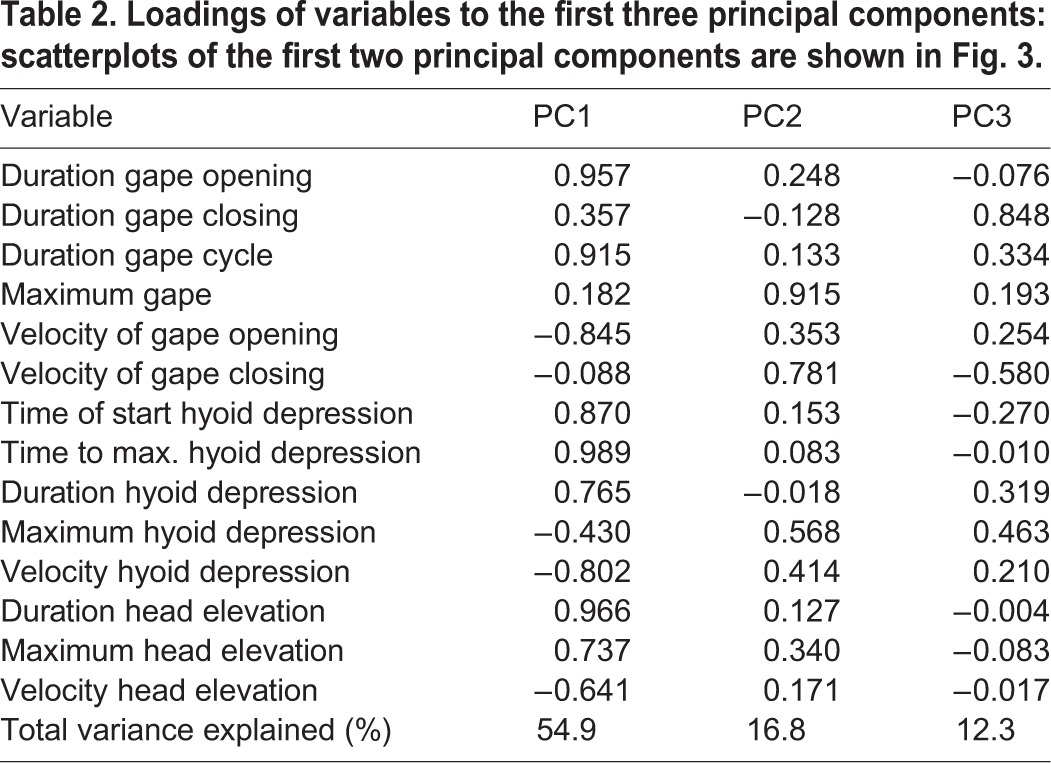
**Loadings of variables to the first three principal components: scatterplots of the first two principal components are shown in Fig. 3.**

#### Variation of behavior

The coefficient of variation (CV) of the kinematic variables was 0.3±0.28 (mean±s.d.) for aquatic and 0.22±0.07 for terrestrial feeding events. The Whitney-U test revealed no significant differences of the CVs between feeding modes (*U*=73; *P*=0.679).

#### Correlation between movements

In aquatic feeding events, significant correlations were found between both the timing (*r*=0.799, *P*<0.001) and the magnitudes of maximum gape and maximum hyoid depression (*r*=0.675, *P*=0.006). In terrestrial capture events, the timing of maximum gape correlated significantly with the timings of maximum hyoid depression (*r*=0.888, *P*<0.001) and maximum tongue protraction (*r*=0.871, *P*<0.001), but no significant correlations were found between the magnitudes of maximum gape and maximum hyoid depression (*r*=0.316, *P*=0.251) or maximum gape and maximum tongue protraction (*r*=0.225, *P*=0.42). All correlations tested are shown in Table 3.

**Table 3.**
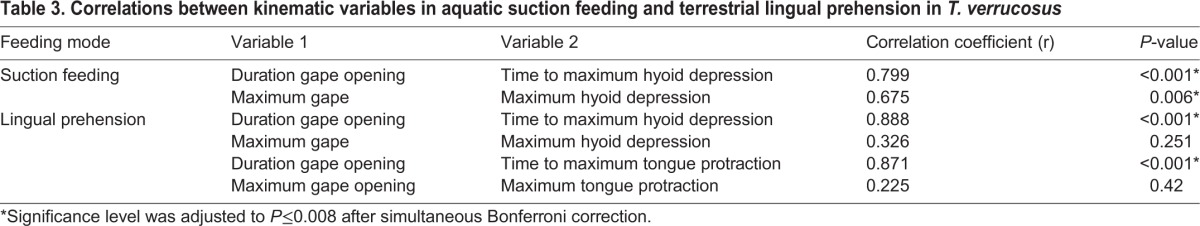
**Correlations between kinematic variables in aquatic suction feeding and terrestrial lingual prehension in *T. verrucosus***

## DISCUSSION

Despite the suggestion by Miller and Larsen (1989) that the Himalayan newts could not be induced to feed in water, we hypothesized that *T. verrucosus* is capable of behavioral flexibility by modifying its prey capture strategy to feed in water. This assumption was based on the observations that: (i) *T. verrucosus* is not exclusively terrestrial but exhibits an aquatic phase at least during its breeding season (Dasgupta, 1996; Thorn, 1968); and (ii) analyses of stomach contents of wild populations revealed that Himalyan newts fed on a variety of aquatic organisms during the monsoon season when they seek aquatic habitats to breed (Dasgupta, 1996). In fact, all animals used in this study regularly sought the aquatic part of their tank and readily fed under aquatic conditions. Accordingly, it might be assumed that *T. verrucosus* also captures prey both on land and in water in its natural habitat. Our observations and kinematic analyses showed that *T. verrucosus* uses a different capture mode in water as used on land. In aquatic conditions, *T. verrucosus* always captured the offered maggots by a fast suction strike. The analyses of the high-speed recordings showed that fast jaw opening, followed by hyoid depression, caused rapid oropharyngeal volume expansion which in turn induced the maggot to accelerate into the gaping mouth (Fig. 1 and 2). The maggot disappeared into the newts' mouth before both gape opening and hyoid depression reached their peaks. Together with the maggot, a considerable amount of water entered the expanded oropharyngeal cavity that was slowly expelled after the strike through the slightly opened gape while the hyoid was elevated. The kinematic pattern for aquatic strikes in *T. verrucosus* with the bell-shaped gape profile, a slightly delayed hyoid depression and prey that is accelerated into the gaping mouth, largely matches the pattern typically found in other suction-feeding salamanders (Deban and O'Reilly, 2005; Heiss et al., 2013a; Lauder and Shaffer, 1985; Reilly, 1995, 1996; Reilly and Lauder, 1992).

Suction feeding is the primary feeding mode in larval salamanders, and post-metamorphic salamanders capable of efficient suction feeding have retained the anatomical and behavioral requirements for suction feeding from the larval condition (Lauder and Gillis, 1997; Lauder and Shaffer, 1988; O'Reilly et al., 2002; Shaffer and Lauder, 1988). Accordingly, also taking into account the comparable level of stereotypy and coordination of movements in the two very different feeding modes (lingual prehension and suction feeding), it is unlikely that the Himalayan newts used in this study have ‘learned’ how to perform suction feeding but that this behavior is still intrinsic for the species. If suction feeding were secondarily acquired by the three individuals tested, more variation between individuals, as well as lower levels in stereotypy and coordination of movements compared to lingual prehension (formerly considered as the primary feeding mode) could be expected. This expectation was based on the following assumptions: first if individuals learn a new behavior independently from each other, the resulting behaviors might differ to a larger degree from each other, compared to inherited behavior; second, if individuals adapt their behavior stepwise to a new situation, there might be higher variation from trial to trial. Higher variation, in turn, results in lower stereotypy (Wainwright et al., 2008) and might result in a lower degree of coordination of movements of mechanically uncoupled elements (i.e. gape and hyobranchial system in *T. verrucosus*) compared to inherited behavior in the same species.

In terrestrial capture events, the movement pattern in *T. verrucosus* was radically different from aquatic strikes. On land, prey was always captured by the quickly protruded adhesive tongue that was subsequently retracted into the mouth with adhering prey (see also Miller and Larsen, 1990). The gape profile was asymmetrical with mouth opening taking considerably longer than mouth closing. This asymmetry is largely based on the fact that tongue pro- and retraction happen during mouth opening and mouth only starts closing once tongue and adhering prey are engulfed. Based on the kinematic profile, the whole gape cycle of terrestrial feeding could be subdivided into four phases: fast opening of the mouth (phase one) is followed by a plateau-like phase (phase two) after which mouth is rapidly opened again and reaches its peak (phase three). After peak gape, mouth is closed (phase four). These four phases correlate with lingual movements as tongue is protracted during phase one and phase two, reaches its peak shortly after start of phase three and is retracted and brought back into the mouth shortly after start of phase four (Fig. 2). Accordingly, the four-phased gape cycle is the result of well-timed tongue and jaw movements which act in concert to allow efficient prey capture by the tongue. In contrast, suction feeding demands a two-phased gape cycle where hyoid starts depressing within phase one (gape opening) and reaches its peak close to the onset of phase two (gape closing).

While two-phased gape cycles are the rule for suction feeding in all salamanders studied so far (Deban and Wake, 2000; Deban and Marks, 2002; Deban and O'Reilly, 2005; Heiss et al., 2013a,b, 2015; Reilly, 1996; Reilly and Lauder, 1989; Shaffer and Lauder, 1988), not all salamanders exhibit a four-phased gape cycle when capturing prey on land with their tongue. Salamanders such as terrestrial feeding ambystomatids have a three-phased gape cycle where the Himalayan newts' phase three (second increase of mouth opening) is lacking (Beneski et al., 1995; Reilly and Lauder, 1989). The four-phased gape cycle is seen in most terrestrial salamanders from different groups (Deban and Marks, 2002; Findeis and Bemis, 1990; Larsen et al., 1996, 1989; Miller and Larsen, 1990; Reilly, 1996) and it had been argued in the past that it is the ancestral condition, whereas the three-phased gape cycle is derived (Lauder and Gillis, 1997). At closer inspection, however, it becomes evident that many terrestrially feeding salamanders from different groups do not use four-phased gape cycles; for example, some plethodontids use two- or three-phased gape cycles when capturing prey by the tongue (Deban and Marks, 2002), and some salamandrids were reported to capture prey on land with their jaws. Jaw prehension for terrestrial prey capture is observed in aquatic salamandrids that occasionally strike on land (Miller and Larsen, 1990) but also in multiphasic newts that capture prey on land in their aquatic (breeding) stage (Heiss et al., 2013a, 2015). The kinematics of the gape cycle of salamanders using jaw prehension to strike prey on land consists of two phases and accordingly differs from the four- and three-phased gape profiles. The movement profile of jaw prehension is similar to the profile observed in aquatic capture events (bell-shaped curve, two phases). Accordingly, we suggest a mechanistic evolutionary scenario where aquatic feeding represents the ancestral pattern retained from the larval condition (Lauder and Gillis, 1997; O'Reilly et al., 2002) and that terrestrial strikes by jaw prehension with a similar profile to aquatic strikes are derived from the aquatic feeding pattern as only little change in movement patterns, and accordingly neuromotor control, is necessary to switch from suction feeding in water to jaw prehension on land (Heiss et al., 2013a, 2015). In a next evolutionary step, prey might have been captured by the pro- and retracted tongue and the kinematic profile became three- or four-phased to coordinate gape and tongue movements accordingly. *T.* *verrucosus* therefore masters its feeding dichotomy by having developed a lingual prehension mode with a four-phased gape cycle but at the same time retained the capability of suction feeding using a two-phased gape cycle.

*T. verrucosus* are comparatively slow feeders both in water and on land (suction feeding: 113.07±18.91 ms versus lingual prehension: 167.47±21.16 ms) when comparing them to aquatic and terrestrial specialists. For example, high-performance suction feeders within ambystomatids (e.g. *Ambystoma mexicanum*, *A. mabeei*), sirenids (e.g. *Siren intermedia*), amphiumids (e.g. *Amphiuma means*), proteids (e.g. *Necturus maculosus*), cryptobranchids (e.g. *Cryptobranchus allenaniensis*), plethodontids (e.g. *Stereochilus marginatus*) or salamandrids (e.g. *Pachytriton* sp., *Ichthyosaura alpestris, Lissotriton vulgaris*) can fulfill their suction-strike in less than 70 ms (Beneski et al., 1995; Deban and Wake, 2000; Deban and Marks, 2002; Heiss et al., 2013a, 2015; Reilly and Lauder, 1992). High-performance lingual feeders within plethodontids (e.g. *Pseudotriton ruber*, *Ensatina eschscholzii*, *Plethodon glutinosus*, *Bolitoglossa occidentalis*), hynobiids (e.g. *Hynobius kimurae*, *H. nebulosus)* and salamandrids (e.g. *Salamandra salamandra*) can accomplish their lingual-based strike within 90-115 ms (Deban and Marks, 2002; Deban and Richardson, 2011; Larsen et al., 1989; Miller and Larsen, 1990). Overall, it might be assumed that the faster a prey can be captured and brought within the margins of the jaws, the fewer time remains for the prey-organism to react to the thread by escape. The time needed to fulfill a capture event might therefore directly influence prey capture success on elusive prey. *T.* *verrucosus* is relatively slow in its prey capture movements but has the advantage of switching between suction feeding in water and lingual prehension on land. This behavioral flexibility allows exploiting food sources from two very different habitats which in turn might increase the energy-intake rate in changing seasonal environmental conditions with changing seasonal prey abundance.

## MATERIALS AND METHODS

### Study animals

*Tylototriton verrucosus* (Anderson, 1871), the Himalayan newt, inhabits high altitudes of the Himalaya region within northeast India, Bhutan, eastern Nepal, North Vietnam and southern China (Khonsue et al., 2010; Stuart et al., 2008). The Himalayan newt predominantly lives in terrestrial habitats during its non-reproductive period but was reported to be partly aquatic during the reproductive period between May and June during the monsoon season (Dasgupta, 1996; Roy and Mushahidunnabi, 2001; Thorn, 1968).

Three adult female Himalayan newts with a mean snout-to-vent length of 73±3 mm (mean±s.d.) and a weight of 15±3 g were used for the present study. The animals were obtained from a commercial breeder and were brought to the Lab of Functional Morphology (University of Antwerp) and kept at room temperature (ca. 20°C) in a 200 liter tank filled with 15 cm of water and an easily accessible land part. Light regime was kept at 12 h light:12 h dark. Animals were fed 3 times a week with maggots (*Lucilia* sp.) earth worms, chironomid larvae, tubifex and fire brads. Animal husbandry and experiments were approved by the Ethical Commission for Animal Experiments of the University of Antwerp (code: 2010-36).

### High-speed video recordings

For high-speed recordings, animals were habituated to feed in a small glass aquarium (20×12×20 cm) where they were recorded from a strict lateral view with a digital high-speed camera (Redlake Motion-Pro HR1000a; Redlake Digital Imaging Systems, IDT Vision, Tallahassee, FL, USA) with a frame rate of 250 Hz. To avoid distortive effects of different prey types on the prey-capture behavior (Deban, 1997; Maglia and Pyles, 1995) we used living maggots (*Lucilia* sp.) as standardized prey items. Maggots were also used because they are a natural prey and all animals showed a strong preference for feeding on them. As a light source, two infrared spotlights were used to minimize the stress load for the newts. A background reference of 20 mm was used for calibration. To document terrestrial feeding, the newts were placed in the empty aquarium and lured with a maggot into the camera's view after which, the maggot was left ∼2 cm in front of the newt and the feeding event was recorded. To document aquatic feeding, maggots were offered in front of the newt in the experimental aquarium with a 5 cm water level. From a total of 70 recorded prey capture events, 5 recordings for each of the 3 individuals for both aquatic and terrestrial prey capture were selected for further analysis. The recordings were selected based on a strict lateral orientation of the newts during prey capture (less than ∼15 degrees of axial rotation of the body) and good visibility of head and trunk (recordings where newts turned their head or moved out of the focus area during a strike were excluded from further analyses). However, all 70 recordings were used for qualitative observations to assess which general capture mode was used in the corresponding medium. The sample size used in this study is comparable with previously published work on feeding kinematics in vertebrates (e.g. Deban, 1997; Deban and Richardson, 2011; Heiss et al., 2013a; Konow et al., 2013; Michel et al., 2015a; Van Wassenbergh, 2013) and therefore deemed appropriate for our approach.

The x-y-coordinates of the eight previously defined anatomical landmarks were tracked frame-by-frame using the open source image processing program ImageJ (National Institutes of Health, USA).

**Fig. 4.**
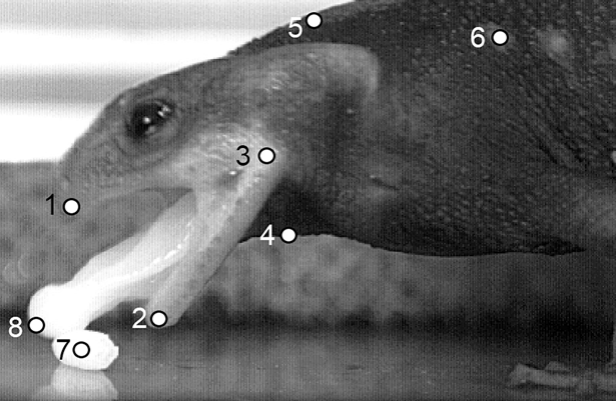
**Anatomical landmarks used for kinematic analyses.** (1) Upper jaw tip; (2) lower jaw tip; (3) jaw joint; (4) hyoid; (5) nape; (6) dorsal trunk reference (first lateral wart); (7) prey (used for aquatic feeding only); (8) tongue tip (used for terrestrial feeding only).

The landmarks largely corresponded with those used in other studies on salamander feeding kinematics (Deban, 1997; Deban and Marks, 2002; Deban and O'Reilly, 2005; Heiss et al., 2013a,b, 2015; Reilly, 1995, 1996; Shaffer and Lauder, 1985) which enabled direct comparison amongst studies. The anatomical landmarks are shown in Fig. 4 and comprised (a) the tip of the upper jaw, (b) the tip of the lower jaw, (c) the jaw joint, (d) the hyoid (floor of mouth), (e) the nape, (f) a dorsal trunk reference (first lateral wart), (g) the prey (only in aquatic feeding events) and (h) the tongue tip (only in terrestrial capture events).

Based on the 2D displacements of the anatomical landmarks, the following kinematic profiles were determined: jaw movement (angle enclosed by the jaws), hyoid depression (distance between jaw joint and hyoid), head rotation (dorsoventral angle displacement of the head relative to the trunk), prey movement (horizontal displacement of the estimated center of mass of the prey; only in aquatic capture events) and tongue movement (tongue pro- and retraction relative to the jaw joint; only in terrestrial capture events).

From these kinematic profiles, twelve kinematic variables that best described the prey capture events were determined: (1) duration of gape cycle (time from start of mouth opening till mouth closure), (2) duration of gape opening (time from mouth opening till peak opening), (3) duration of gape closure (time from peak mouth opening till closure), (4) maximum gape (maximum angle between upper and lower jaw shafts minus initial value), (5) average angular velocity of gape opening (maximum gape angle divided by duration of mouth opening), (6) average angular velocity of gape closing (maximum gape angle divided by duration of mouth closing), (7) time to start of hyoid depression (time from start of mouth opening to start of hyoid depression), (8) time to maximum hyoid depression (time from start of gape cycle to maximum hyoid deflection), (9) duration of hyoid depression (time from start of hyoid depression to maximum hyoid deflection), (10) maximum hyoid depression (maximum distance between jaw joint and hyoid minus initial value), (11) average velocity of hyoid depression (maximum hyoid depression divided by duration of hyoid depression), (12) duration of head elevation (time from start of dorsal head rotation to maximum elevation), (13) maximum head elevation (maximum angle of head relative to trunk minus initial value), (14) average angular velocity of head elevation (maximum angle of head elevation divided by duration of head elevation).

### Statistics

After calculating descriptive statistics for each kinematic variable and individual, homogeneity and normal distribution of the variables' residuals were tested. Residuals in an ANOVA in general are the distance of observed values to the group or (factor level) mean, i.e. the difference between the mean and the observed value of the independent variable. Residuals were calculated for each of the independent variables in the MANOVA and the means of each combination of the fixed factors used to generate the distances. As all residuals were homogenous and normally distributed, a multivariate analysis of variance (MANOVA) was performed where both ‘individual’ and ‘feeding mode’ were treated as fixed factors and the fourteen variables as random effects. To account for running multiple tests (i.e. the subsequent series of ANOVAs), the simultaneous Bonferroni correction was used to adjust significance levels to *P*≤0.0038 for all resulting ANOVAs. Next, a principal component analysis was performed to show the effects of (a) individual and (b) feeding mode on the total variance.

To test for behavioral variation between aquatic and terrestrial feeding modes, the coefficient of variation (CV) was calculated as standard deviation divided by mean values (Wainwright et al., 2008) for each of the 14 kinematic variables for both feeding modes. As the residuals of the CVs did not conform to the requirements for parametric tests, we performed the nonparametric Whitney-U test to unravel differences between feeding modes.

The coordination between movements was calculated as bivariate (Pearson) correlations between kinematic variables (Wainwright et al., 2008). We tested for correlations between gape and hyobranchial (including the tongue) movements because gape and hyobranchial movements are not mechanically coupled (Deban and Wake, 2000; Wake and Deban, 2000) and the correlation of their variables is a good indicator for active coordination (Wainwright et al., 2008). To account for multiple correlations, significance level was adjusted to *P*≤0.008 after simultaneous Bonferroni correction.

Statistical analyses were performed with Microsoft Excel 2010 (Microsoft, Redmond) and SPSS Statistics 22 (IBM, Armonk) software package.
